# Age and product dependent vaccine effectiveness against SARS-CoV-2 infection and hospitalisation among adults in Norway: a national cohort study, July–November 2021

**DOI:** 10.1186/s12916-022-02480-4

**Published:** 2022-09-02

**Authors:** Jostein Starrfelt, Anders Skyrud Danielsen, Eirik Alnes Buanes, Lene Kristine Juvet, Trude Marie Lyngstad, Gunnar Øyvind Isaksson Rø, Lamprini Veneti, Sara Viksmoen Watle, Hinta Meijerink

**Affiliations:** 1 Department of Infection Control and Preparedness, Norwegian Institute of Public Health, Postboks 222 Skøyen, 0213 Oslo, Norway; 2 Department of Microbiology, Oslo University Hospital, Oslo, Norway; 3 Norwegian Intensive Care and Pandemic Registry (NIPaR), Helse Bergen Health Trust, Bergen, Norway; 4 Department of Anaesthesiology and Intensive Care Haukeland University Hospital, Bergen, Norway; 5 Department of Infection Control and Vaccines, Norwegian Institute of Public Health, Oslo, Norway; 6 Department of Development and Analytics, Norwegian Institute of Public Health, Oslo, Norway

**Keywords:** COVID-19, SARS-CoV-2, COVID-19 vaccines, Vaccine effectiveness, Proportional hazards models, Intensive care units, Mortality, Hospitalization, Cohort studies, Vaccination, Norway, Registries

## Abstract

**Background:**

COVID-19 vaccines have been crucial in the pandemic response and understanding changes in vaccines effectiveness is essential to guide vaccine policies. Although the Delta variant is no longer dominant, understanding vaccine effectiveness properties will provide essential knowledge to comprehend the development of the pandemic and estimate potential changes over time.

**Methods:**

In this population-based cohort study, we estimated the vaccine effectiveness of Comirnaty (Pfizer/BioNTech; BNT162b2), Spikevax (Moderna; mRNA-1273), Vaxzevria (AstraZeneca; ChAdOx nCoV-19; AZD1222), or a combination against SARS-CoV-2 infections, hospitalisations, intensive care admissions, and death using Cox proportional hazard models, across different vaccine product regimens and age groups, between 15 July and 31 November 2021 (Delta variant period). Vaccine status is included as a time-varying covariate and all models were adjusted for age, sex, comorbidities, county of residence, country of birth, and living conditions. Data from the entire adult Norwegian population were collated from the National Preparedness Register for COVID-19 (Beredt C19).

**Results:**

The overall adjusted vaccine effectiveness against infection decreased from 81.3% (confidence interval (CI): 80.7 to 81.9) in the first 2 to 9 weeks after receiving a second dose to 8.6% (CI: 4.0 to 13.1) after more than 33 weeks, compared to 98.6% (CI: 97.5 to 99.2) and 66.6% (CI: 57.9 to 73.6) against hospitalisation respectively. After the third dose (booster), the effectiveness was 75.9% (CI: 73.4 to 78.1) against infection and 95.0% (CI: 92.6 to 96.6) against hospitalisation. Spikevax or a combination of mRNA products provided the highest protection, but the vaccine effectiveness decreased with time since vaccination for all vaccine regimens.

**Conclusions:**

Even though the vaccine effectiveness against infection waned over time, all vaccine regimens remained effective against hospitalisation after the second vaccine dose. For all vaccine regimens, a booster facilitated recovery of effectiveness. The results from this support the use of heterologous schedules, increasing flexibility in vaccination policy.

**Supplementary Information:**

The online version contains supplementary material available at 10.1186/s12916-022-02480-4.

## Background

Since the start of the COVID-19 pandemic, various COVID-19 vaccines have been approved for Emergency Use Listing/Authorization (EUL/EUA), including Comirnaty (Pfizer/BioNTech; BNT162b2), Spikevax (Moderna; mRNA-1273), Vaxzevria (AstraZeneca; ChAdOx nCoV-19; AZD1222), and Janssen (Johnson & Johnson; Ad26.COV2.S). Both vaccine efficacy estimates from randomised controlled trials and vaccine effectiveness estimates from observational studies in the first months after the vaccine roll-out showed strong protection against both infection and severe disease [1–6]. However, effectiveness may differ between product types and against different virus variants, as well as be affected by dose intervals or population structure (age distribution, risk groups) [7–13]. Many countries have adopted flexible policies allowing “mixing and matching” of vaccines (heterologous regimens) during SARS-CoV-2 vaccination campaigns, in response to supply constraints, policy changes, or rare but severe side effects associated with the vector-based vaccines [14–16]. Combining the vector-based vaccines, such as Vaxzevria, with an mRNA vaccine increases the vaccine effectiveness to a level comparable with mRNA regimens [7, 17–19]. However, a possible waning of vaccine-induced immunity could result in lower vaccine effectiveness over time [20, 21]. A systematic review of the recently published data found reduced protection against SARS-CoV-2 infection in all age groups 6 months after the completion of a primary vaccination regimen and, also, a small decrease against severe disease in certain groups [22].

On 27 December 2020, Norway started COVID-19 vaccination, initially targeted towards elderly (> 65 years) and risk groups. Of those ≥ 18 years, 88% had received at least two vaccine doses by 5 December 2021. Vaxzevria was included in the Norwegian national vaccine programme until 11 March 2021; those who received one dose were offered a second dose with an mRNA vaccine. Since September 2021, a booster dose has been recommended, initially prioritising those above 65 years and risk groups, including health care workers. From early February 2021, the Alpha variant (B1.1.7) was the dominant circulating strain in Norway, being replaced by the Delta variant (B.1.617.2) by July and Omicron by December 2021 [10, 12, 23, 24]. During the period, when the Delta variant was dominant, SARS-CoV-2 PCR testing was widely available and free for anyone, including but not limited to those with mild symptoms, risk groups, and close contacts. In addition, all positive rapid test were confirmed by SARS-CoV-2 PCR.

Understanding the changes in vaccines effectiveness over time, the impact of giving boosters, and differences between vaccine types is essential to guide vaccine implementation and policies. Even though the Delta variant is no longer dominant in many regions, understanding these properties will provide essential knowledge that can be used to understand the development of the pandemic and estimate potential changes over time. The purpose of this study is to quantify and compare the vaccine effectiveness against infection, disease, and death achieved in the Norwegian population during the Delta epidemic considering time since vaccination, vaccine type, and age groups.

## Methods

### Study population

For this population-based cohort study, we linked data from the Norwegian National Preparedness Register for COVID-19 (Beredt C19) (Additional file 1: Table S1). This register is a “data lake” in which several different publicly owned data sources, like the central health registers and national administrative registers, are gathered and made available to the Norwegian Institute of Public Health. All residents in Norway have a unique personal identification number. This repository allows for the real-time surveillance and analysis of all individual-level data relating to the pandemic. For this study, we extract and link data from six different sources—the National Population Registry (NPR), The Norwegian Immunisation Register (SYSVAK), The Norwegian Intensive Care and Pandemic Registry (NIPaR), The Norwegian Surveillance System for Communicable Diseases (MSIS), Statistics Norway (SSB), and an internally created table of risk group membership. These registers include and cover the entire Norwegian population, and the reporting of testing, test results, vaccinations, hospitalisations, and mortality are mandatory by law and considered complete. We included all adults (≥ 18 years by the end of 2021) with a valid national identity number and registered in the NPR as living in Norway. To remove non-standard vaccination histories, we removed individuals with more than three doses before the end of the study period and excluded individuals for which the interval between first and second dose was shorter than the recommended minimum intervals and censored those with a third dose registered before the recommended 120 days of the second dose. The recommended minimum interval between first and second dose was based on the vaccine type given as the first dose; 19 days for Comirnaty, 22 days for Spikevax, and 21 days for Vaxzevria. We only included individuals who had received either of the three vaccines that are part of the Norwegian vaccination programme (Comirnaty, Spikevax or Vaxzevria). Finally, individuals admitted to hospital with COVID-19 without a corresponding match in the database for the time of positive test were excluded. Data were extracted from the registries on 15 February 2022.

### Definitions

SARS-CoV-2 infection is defined as a positive SARS-CoV-2 PCR test reported to the MSIS registry, which has been complete for all domestic laboratories since April 2020. Individuals who were hospitalised or admitted to an intensive care unit (ICU) with COVID-19 are registered in NIPaR, which covers mandatory reporting from all Norwegian hospitals [25]. We included all hospitalisation where COVID-19 was registered as the primary diagnosis for admission and ICU admission of individuals who tested positive for SARS-CoV-2 and were admitted to an ICU (length of stay ≥ 24 h), required mechanical ventilatory support (invasive or non-invasive), or persistent administration of vasoactive medication. All COVID-19-associated deaths are defined as anyone with a positive SARS-CoV-2 PCR test who died with COVID-19 reported on the death certificate in the Cause of Death Register (DÅR) or those notified directly to MSIS. We use testing date as time of infection (positive PCR test) and vaccination status is determined at time of infection for all outcomes. Individual vaccination histories were generated from SYSVAK and categorised into the following vaccination statuses:

- Unvaccinated: unvaccinated up to seven days before the first dose
- 1st dose: ≥ 21 days after first vaccine dose up to 7 days after second vaccine dose
- 2nd dose: > 7 days after the 2nd dose, divided in period of 8 weeks
- 3rd dose (booster): > 7 days after a vaccine dose given 120 days or more after completion of the primary vaccine regimen

The vaccine regimens included were Comirnaty, Spikevax, heterologous mRNA regimen, Vaxzevria, or Vaxzevria in combination with an mRNA vaccine, all with or without an mRNA booster. The period between seven days before the first vaccine dose until 21 days after the first dose was included as a separate status not reported, since vaccination was postponed if individuals showed signs of infection which could potentially bias infection rates for both unvaccinated and partially vaccinated if included in the adjacent vaccination statuses. Similarly, individuals were also included as a separate status and not reported for the first 7 days after receiving the third dose. Individuals with a SARS-CoV-2 infection registered prior to 1 June 2021 were included as a separate category (see below).

To adjust for confounding, several covariates were included in our analysis. When stratifying by age, we used the categories 18 to 44 years, 45 to 64 years, and 65 years or older. For adjustment in other models, we used 10-year age bands (NPR). County of residence (NPR) was included as both infection rates and speed of vaccine rollout has varied across Norway. We included country of birth (NPR; Norway, abroad or unknown) and crowded living conditions (SSB; crowded, not crowded, or unknown) since both are associated with vaccine coverage and risk of infection. Individuals with pre-existing medical conditions associated increased risk of severe COVID-19 illness were prioritised for vaccination and this covariate was also included in the adjusted model. Missing values were considered as a separate category for each of the variables where relevant. More details on the data sources and variables can be found in the supplementary information as well as the number of infections, hospitalisations, and vaccination status over time (Additional file 1: Table S1, Fig. S1 and Fig. S2).

### Data analyses

We estimated the vaccine effectiveness using Cox proportional hazards models on an open cohort, using vaccine status as time-varying covariate for all individuals included in the statistical software *R* [26]. We included all SARS-CoV-2 infections reported from 15 July until 30 November 2021, the period in which the delta variant was dominating in Norway [10]. We right-censored individuals at the time of an event (SARS-CoV-2 infection, hospitalisation, ICU admission, or death associated with COVID-19), time of death (all cause), or end of follow-up period (30 November 2021). During the study period, the registries in Norway only report re-infections if 6 months or more since last positive test, individuals registered with an infection prior to 15 July 2021 entered the dataset 180 days since positive test with status ‘previously infected’.

Vaccine effectiveness is defined as 100*(1–*β*), where *β* represents the hazard ratio associated with a particular vaccine status. For crude vaccine effectiveness estimates, we only used vaccine status as a time-varying covariate (supplementary analyses; Additional file 1: Tables S2-S5). For adjusted estimates, we implemented stratified analyses using *strata(variable)* in the *survival-*package [27], i.e. that the impact of the adjustment variables can be non-proportional.

To estimate specific vaccine effectiveness for age groups and vaccine product regimens, we use independent Cox-models while still adjusting for the remaining covariates as *strata*. Vaccine status was factored either by combining all vaccine types, including Comirnaty, Spikevax, and Vaxzevria (thus assuming similar effectiveness across vaccines) to estimate a population level vaccine effectiveness of the vaccination programme in Norway or by implementing vaccine status as the combination of vaccine type and vaccine status. Due to the smaller numbers and partial exclusion of Vaxzevria from the vaccination programme in Norway, all individuals who received a dose of Vaxzevria were censored at time of the dose in the product-specific analyses. Models were also run excluding all unvaccinated individuals who have never had a recorded SARS-CoV-2 PCR test in Norway, results from these models can be found in the supplementary materials (Additional file 1: Tables S2-S6).

## Results

Of the 4,310,345 individuals in the collated dataset, we excluded 8350 individuals. Specifically, we excluded 1348 individuals with more than three doses, 678 individuals with shorter than the recommended minimum intervals between vaccine doses, 6257 individuals who received other vaccines, and 67 individuals admitted to hospital with COVID-19 without corresponding positive test. In addition, 17,497 individuals were censored with a third dose registered before the recommended 120 days of the second dose.

Amongst 4,301,995 individuals included (99.8%), 75,303 were diagnosed with SARS-CoV-2 infection, 1438 were hospitalised with COVID-19 as main cause for admission, 289 were admitted to the ICU, and 331 died with COVID-19 between 15 July and 30 November 2021. Characteristics of the study population and by outcome can be found in Table 1. Overall vaccine effectiveness against infection was estimated at 24.7% (confidence interval/CI: 22.7 to 26.7) after the first dose, 65.2% (CI: 64.6 to 65.9) after the second dose and 84.8% (CI: 83.3 to 86.3) after the third dose. Having a documented infection > 180 days prior reduced the probability of infection by 93.5% (CI: 92.7 to 94.2).

**Table 1:** Characteristics of study population and by outcomes of interests; SARS-CoV-2 infections, hospitalisation, intensive care unit (ICU) admission, and death in Norway, 15 July–30 November 2021

		Total study population		SARS-CoV-2 infection		Hospitalisation		ICU admission		COVID-19 deaths	
		***n***	%	***n***	%	***n***	%	***n***	%	***n***	%
**All**	**-**	4,301,995	-	74,371	-	1429	-	290	-	331	-
**Age groups**	18–24 years	457,238	10.6	11,499	15.5	24	1.7	1	0.3	0	0.0
	25–34 years	747,249	17.4	16,013	21.5	113	7.9	16	5.5	2	0.6
	35–44 years	705,460	16.4	17,131	23.0	190	13.3	40	13.8	2	0.6
	45–54 years	735,420	17.1	14,050	18.9	206	14.4	46	15.9	6	1.8
	55–64 years	653,259	15.2	7440	10.0	202	14.1	63	21.7	22	6.6
	65–74 years	540,898	12.6	4604	6.2	214	15.0	52	17.9	38	11.5
	75–84 years	335,628	7.8	2557	3.4	301	21.1	59	20.3	103	31.1
	≥ 85 years	126,843	2.9	1077	1.4	179	12.5	13	4.5	158	47.7
**Sex**	Male	2,160,307	50.2	37,098	49.9	791	55.4	196	67.6	185	55.9
	Female	2,141,688	49.8	37,273	50.1	638	44.6	94	32.4	146	44.1
**Underlying conditions**	No risk group	3,401,381	79.1	62,802	84.4	711	49.8	129	44.5	78	23.6
	Medium risk	788,954	18.3	10,247	13.8	524	36.7	115	39.7	180	54.4
	High risk	111,660	2.6	1322	1.8	194	13.6	46	15.9	73	22.1
**Country of birth**	Norway	3202,876	74.5	46,501	62.5	791	55.4	170	58.6	247	74.6
	Outside Norway	802,615	18.7	25,579	34.4	506	35.4	103	35.5	34	10.3
	Unknown	296,504	6.9	2291	3.1	132	9.2	17	5.9	50	15.1
**Crowding**	Yes	336,777	7.8	11,953	16.1	175	12.2	29	10.0	7	2.1
	No	3,728,263	86.7	57,206	76.9	1154	80.8	238	82.1	299	90.3
	Unknown	236,955	5.5	5212	7.0	100	7.0	23	7.9	25	7.6

### Vaccine effectiveness since time of vaccination

**Fig. 1:**
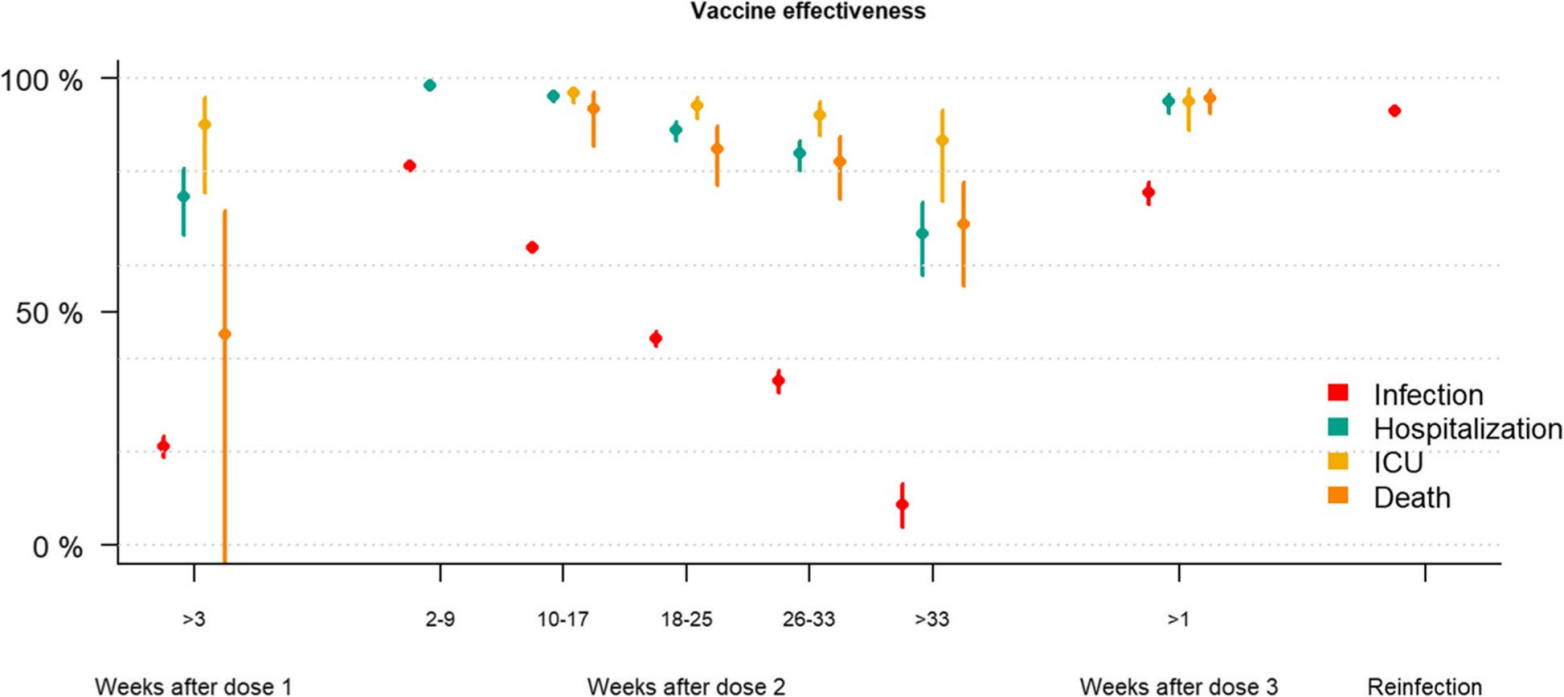
Adjusted vaccine effectiveness against infection (red), hospitalisation (blue), ICU admission (yellow) and COVID-19 associated deaths (orange) for Norwegian adults using data from 15 July to 30 November 2021. Adjusted for age, sex, comorbidities, county of residence, country of birth, and living conditions

The adjusted vaccine effectiveness against infection was high in the first period (2 to 9 weeks) after the second dose (81.3%, CI: 80.7 to 81.9). Effectiveness waned with time since vaccination and more than 33 weeks after receiving the second dose the effectiveness against infection was 8.6% (CI: 4.0 to 13.4). Similarly, the effectiveness against hospitalisation was 98.6% (CI: 97.5 to 99.2) in the period right after receiving the second dose, decreasing to 66.6% (CI: 57.9 to 73.6) after more than 33 weeks. For admission to ICU and death, not enough events in the first period following the second dose had occurred to reliably estimate a vaccine effectiveness, but in the following period (10 to 17 weeks), vaccine effectiveness was 96.9% (CI: 94.7 to 98.1), and 93.4% (CI: 85.4 to 97.0) against ICU and death respectively, with a less pronounced reduction over time for ICU admissions (86.7%, CI: 73.9 to 93.2 after more than 33 weeks) than for death (68.6%, CI: 55.4 to 77.9). One dose provided little protection against infection (30.0%, CI: 28.2 to 31.9) but did protect against hospitalisation (79.4%, CI: 73.0 to 84.2) and ICU admission (92.4%, CI: 80.9 to 97.0). The estimates against death were should not be used for inference as they have wide confidence intervals (46.9%, CI: − 0.2 to 71.9), likely due to small sample. The vaccine effectiveness against infection after receiving a third dose (75.9%, CI: 73.4 to 78.1) was similar to the effectiveness in the initial two to nine weeks after the second dose (81.3%, CI: 80.7 to 81.9), albeit based on a relatively small sample (Additional file 1: Table S2) with short follow-up time (median: 13 days, Q1–Q3: 6–20 days). For those with a previous reported infection (> 6 months prior), the protection against infection was 93.1% (CI: 92.3 to 93.9), whereas too few events amongst those with a reported prior infection were reported to estimate effectiveness against hospitalisation. Vaccine effectiveness for all outcomes split by time is shown in Fig. 1 (details can be found in Additional file 1: Tables S2-S5). The vaccine effectiveness estimates were used to estimate the overall cohort-wide level of protection against SARS-CoV-2 infection over time, showing the overall impact of waning and booster doses (Additional file 1: Fig. S3).

### Vaccine effectiveness by age

**Fig. 2:**
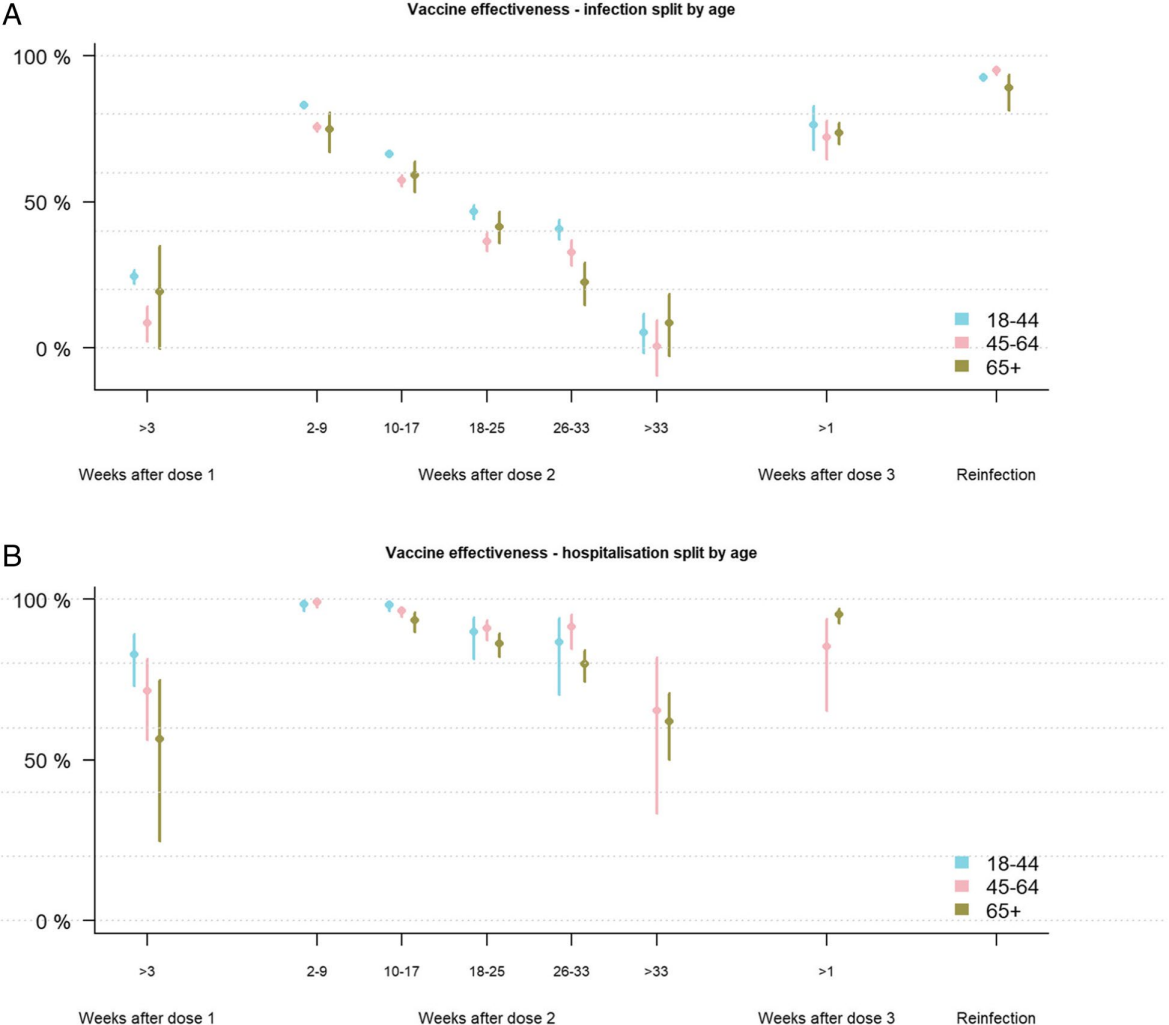
Adjusted vaccine effectiveness against infection (**A**) and hospitalisation (**B**) for age 18–44 years (blue), 45 to 64 (pink) and 65+ years (green) amongst Norwegian adults using data from 15 July–30 November 2021. Adjusted for age, sex, comorbidities, county of residence, country of birth, and living conditions

Vaccine effectiveness against infection was highest in 2 to 9 weeks after the second dose amongst 18- to 44-year-olds (83.2%, CI: 82.6 to 83.8) compared to 45- to 64-year-olds (75.6%, CI: 74.1 to 77.0) and those over 64 years (74.9%, CI: 67.2 to 80.7). No significant protection was found more than 33 weeks after the second dose: the estimated effectiveness against infection was 5.2% (CI: − 1.9 to 11.8) for 18- to 44-year-olds, 0.5% (CI: − 9.4 to 9.5) amongst 45- to 64-year-olds, and 8.4% (CI: − 2.8 to 18.5) amongst those over 65 years (Fig. 2, Additional file 1: Table S6). Amongst those with a reported prior infection, protection against infection was 92.6% (CI: 91.5 to 93.5), 95.1% (CI: 93.6 to 96.3), and 89.0% (CI: 81.3 to 93.5) for 18- to 45-year-olds, 45- to 64-year-olds, and over 65-year-olds respectively (Fig. 2). The protection against hospitalisation was already significant after one dose in all age groups: 82.9% (CI: 73.0 to 89.2) amongst 18- to 44-year-olds, 71.4% (CI: 56.2 to 81.4) amongst 45- to 64-year-olds, and 56.5% (CI: 24.6 to 74.9) amongst those over 65 years. The effectiveness against hospitalisation decreased less with time than protection against infection (Fig. 2). For 45- to 64-year-olds the effectiveness was 99.1% (CI: 97.7 to 99.6) in 2 to 9 weeks after the second dose, compared to 65.3% (CI: 33.3 to 82.0) more than 33 weeks after the second dose. Similarly, amongst those above 65 years, the protection waned from 93.6% (CI: 89.9 to 96.0) to 61.9% (CI: 50.1 to 70.9) (Fig. 2, Additional file 1: Tables S6 - S7). Receiving a third dose increased vaccine effectiveness against hospitalisation to 85.4% (CI: 65.3 to 93.9) amongst 45- to 64-year-olds and 95.3% (CI: 92.6 to 97.0) for over 65-year-olds; the number of events were too small for 18- to 44-year-olds (Fig. 2, Additional file 1: Tables S6 - S7).

### Vaccine effectiveness by product regimen

**Fig. 3:**
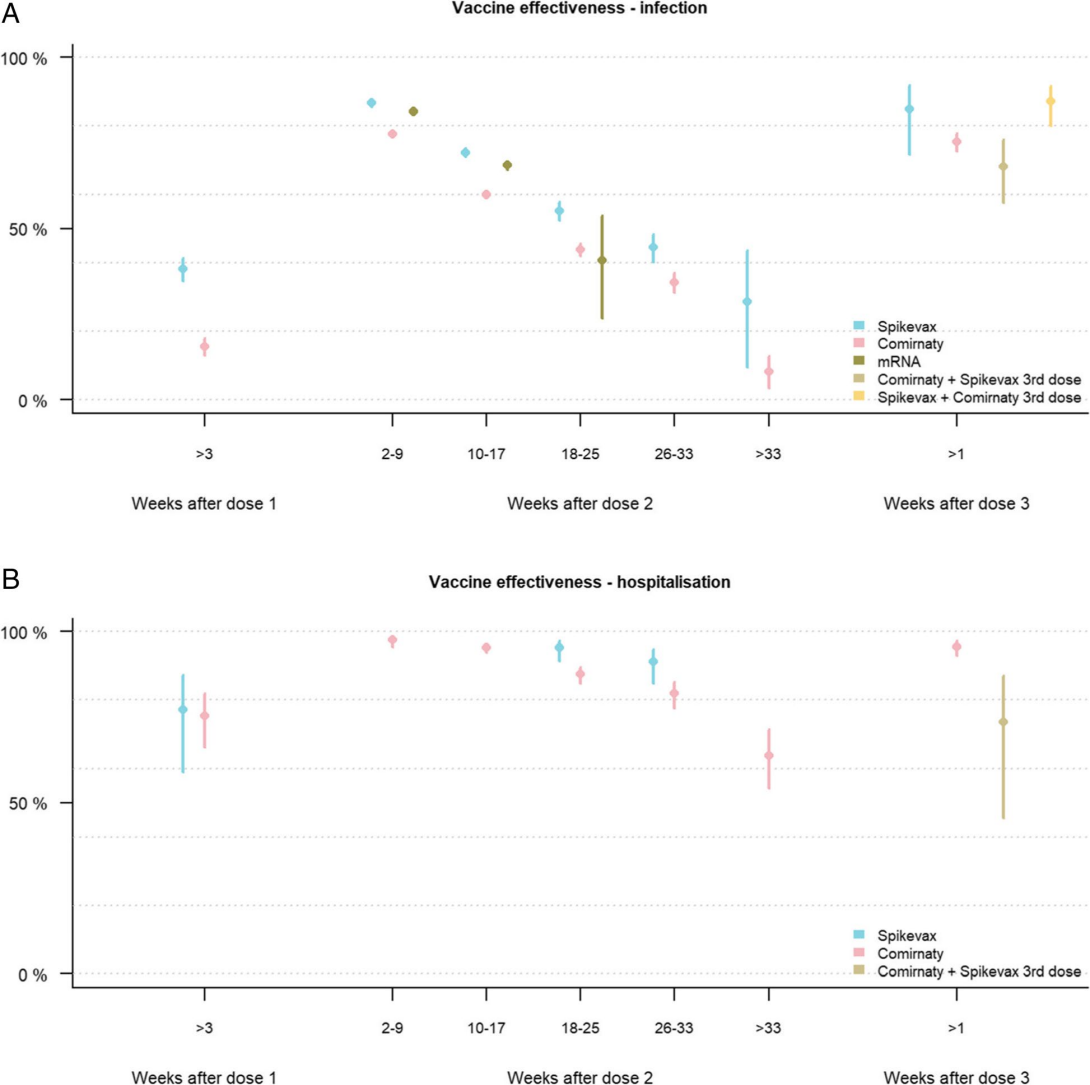
Adjusted vaccine effectiveness against infection (**A**) and hospitalisation (**B**) per vaccine product regimen (Spikevax (blue), Comirnaty (pink), or mixed mRNA primary regimen (dark green) and mixed booster (light green and yellow) amongst Norwegian adults using data from 15 July to 30 November 2021. Adjusted for age, sex, comorbidities, county of residence, country of birth and living conditions. mRNA includes a combination of one dose Spikevax and one dose Comirnaty; only few individuals who received a heterologous primary regimen were eligible for a booster during the study period and are therefore not included

When stratifying by product regimen, individuals who received two doses of Spikevax (86.6%, CI: 85.6 to 87.6) or a heterologous mRNA regimen (84.1%, CI: 83.2 to 85.0) had a higher estimated vaccine effectiveness against infection than those who received two doses Comirnaty (77.7%, CI: 76.8 to 78.5) in 2 to 9 weeks after the second dose (Fig. 3, Additional file 1: Table S8). All product regimens showed waning of vaccine effectiveness against infection (Fig. 3). The vaccine effectiveness against infection after receiving the third dose was highest for those who received two doses of Spikevax followed by a booster with Comirnaty (87.1%; CI: 80.1 to 91.6) or Spikevax (84.9%; CI: 71.8 to 91.9), compared to 75.3% (CI: 72.5 to 77.8) and 68.2% (CI: 57.6 to 76.1) for those who received two doses of Comirnaty followed by a booster with Comirnaty or Spikevax respectively. The vaccine effectiveness against hospitalisation was high after one dose (77.1% and 75.3%) for both Spikevax and Comirnaty, as well as one to 32 weeks after the second dose (range 81.8 to 97.5%). There were only five hospital admissions amongst those who received heterologous mRNA vaccination during our study period and therefore vaccine effectiveness against hospitalisation since time of vaccination could not be estimated. Amongst those who received a booster dose, vaccine effectiveness against hospitalisation could only be estimated for those with primary regimen of Comirnaty and protection was high 95.6% (CI: 93.1 to 97.2) for those receiving a Comirnaty booster and slightly lower but more uncertain for those receiving Spikevax (73.5%, CI: 45.7 to 87.1) (Fig. 3, Additional file 1: Table S9). Supplementary analyses split by age and product regimen showed similar trends (Additional file 1: Fig. S4).

## Discussion

Our analyses showed strong protection against SARS-CoV-2 infection in the first period after two or more vaccine doses for all regimens, including heterologous mRNA regimens. However, for all product regimens and age groups, the vaccine effectiveness against infection waned over time. Vaccine effectiveness against hospitalisation was high for all product regimens and age groups, with limited waning with time since vaccination.

Our findings are consistent with other studies investigating waning of vaccine effectiveness, as summarised through a systematic review by Feikin et al. [22] Most observational studies employ a test-negative study design, while some are register-based cohort studies like ours [20, 28–36]. We reported large waning of effectiveness against SARS-CoV-2 infection, which could have several explanations. First, we have a longer follow-up time than most previously published studies. Second, we use data from a high-quality population register covering an entire national population in a country with widespread and free testing irrespective of symptoms. After vaccination, individuals might modify their behaviour based on their evaluation of risk and change propensity for testing, or unvaccinated could become indirectly protected by an increasing population immunity. In addition, unrecorded prior infections amongst unvaccinated may reduce the detected risk amongst unvaccinated. This is supported by the fact that excluding those reported as unvaccinated who have never been tested resulted in higher vaccine effectiveness for all outcomes (Additional file 1: Tables S2, S3, S4 and S5, subcohort 1), which makes our estimates conservative. Our findings are in line with immunological findings suggesting that antibody titres wane over time [21, 37]. Even though antibody-mediated immunity may wane and require time to reactivate upon infection, the fact that vaccine effectiveness against severe disease remains high is consistent with the induction of cell-mediated immunity [38]. While groups at risk for more severe outcome form a disproportionate part of those hospitalised, admitted to ICU or death (Table 1), an analysis of a subcohort excluding all risk groups showed similar estimates of vaccine effectiveness (Additional file 1: Tables S2, S3, S4 and S5, subcohort 2), which indicates that the vaccines reduce the probabilities of infection and hospitalisation similarly across risk-groups.

To our knowledge, this is amongst the few studies to report on the vaccine effectiveness of heterologous mRNA vaccine regimens. As heterologous vaccine regimens were accepted in Norway from June 2021, recipients of a combination of mRNA vaccines are predominantly younger and healthier. However, these results are also maintained across age groups and are in line with a test-negative case control study from Canada [39]. In addition, we show that Spikevax shows a slightly higher vaccine effectiveness against infection than Comirnaty, as has also been reported by others [9, 40, 41].

During our study period, many aspects of the pandemic changed, and changes also occurred simultaneously. Even though we attempt to control for confounding in the analyses, residual confounding cannot be completely dismissed. Furthermore, testing intensity changed over time, especially with the introduction of self-administered rapid antigen tests after last summer. Even though testing capacity is high in Norway, all individuals infected with SARS-CoV-2 have not been detected, which could affect the estimates since the proportion of unidentified cases may differ depending on age as well as on vaccine status [42]. Additionally, the estimated vaccine effectiveness can be affected by number and types of contacts [43]. As previously mentioned, the vaccine effectiveness could be underestimated if getting vaccinated results in behavioural changes associated with higher risk of exposure. We are not able to incorporate behavioural changes in our analyses. Nevertheless, the estimated population-level effects can provide a reasonable estimate of the individual-level vaccine efficacy since the total attack rate during the study period was small [43].

The ability to link data collected via national registries is a great advantage and allows us to estimate population-wide vaccine effectiveness. However, some limitations of register-based data should be considered when interpreting the results. Data in these registries are not collected for the purpose of this study, and therefore, the focus on level of detail, error checking, and precision in the available data is not guided by the current study, as would be the case for independent data gathering. For instance, while vaccines administered as part of the Norwegian vaccination programme should be in our dataset, it is not unlikely that some have received vaccines outside Norway and not reported them to the Norwegian register (SYSVAK). While limitations in register-based data are important caveats, our cohort study encompassing the whole Norwegian adult population indicates that vaccine effectiveness against severe disease is high amongst vaccinated individuals. Our estimates remain qualitatively the same for protection against infection and severe disease when splitting by age groups, indicating that the confounding effect of factors that are relatively constant within age-groups introduce little bias in our adjusted models.

Appropriate prioritisation and planning of vaccine campaigns is integral for combating COVID-19 and is only possible with updated knowledge on vaccine effectiveness of realistic vaccination regimens achieved in large populations. For our study, the overall protection (i.e. a weighted mean of vaccine effectiveness over time) increases through the initial period with a peak of right below 60% on the 21st of September (Additional file 1: Fig. S3). Coupling VE-estimates with cohort fractions over time can yield valuable information on the general level of protection in a population and timing and prioritisation of vaccine roll-out. Our study amongst adults in Norway indicate a high vaccine effectiveness against both infection and hospitalisation with both homologous and heterologous mRNA regimens. Even though the effectiveness against infection declines with time since vaccination, the protection against severe disease remained high. The results support the use of heterologous regimens, increasing flexibility in vaccination policy.

## Conclusions

During the Delta-phase of the COVID-19 epidemic in Norway, vaccine effectiveness against infection clearly waned over time; however, all vaccine regimens remained effective against hospitalisation after the second vaccine dose. For all vaccine regimens, a booster facilitated recovery of effectiveness. The results from this support the use of heterologous schedules, increasing flexibility in vaccination policy.

## Supplementary Information

**Additional file 1. **Details on the data sources and variables as well as the epidemiological situation in Norway during the study period. In addition, this additional file contains all numbers underlying the visual representation in this manuscript and additional sensitivity analyses performed, such as unadjusted estimates, and estimated on sub-cohorts. Additional File 1: Tables S1-S9 and Figs. S1-S4. **Table S1.** Description of data sources and variables used in this study. **Table S2.** Vaccine effectiveness against SARS-CoV-2 infection for all adults (18+) in Norway. **Table S3.** Vaccine effectiveness against hospitalisation for all adults (18+) in Norway. **Table S4.** Vaccine effectiveness against ICU admission for all adults (18+) in Norway. **Table S5.** Vaccine effectiveness against mortality for all adults (18+) in Norway. **Table S6.** Adjusted vaccine effectiveness against SARS-CoV-2 infection by age groups (18-44, 45-64 and ≥65 years) in Norway. **Table S7.** Adjusted vaccine effectiveness against hospitalisation by age groups (18-44, 45-64 and ≥65 years) in Norway. **Table S8.** Adjusted vaccine effectiveness against SARS-CoV-2 infection and hospitalisation by vaccine product type. **Table S9.** Adjusted vaccine effectiveness against SARS-CoV-2 infection and hospitalisation after receiving a booster dose by vaccine product type. **Fig. S1.** Daily reported cases of SARS-CoV-2 infections and number of hospitalisations, and weekly admissions to ICU and deaths in Norway during the study period, 15 July -30 November 2021. **Fig. S2.** Distribution of vaccine statuses over time in the study population, 15 July–30 November 2021. **Fig. S3.** Cohort-wide level of protection against infection over time based on vaccine coverage and vaccine effectiveness estimates. **Fig. S4.** Vaccine effectiveness against infection and hospitalisations for different vaccine products and combinations of booster doses when splitting the cohort by age.

**Additional file 2.** STROBE check list.

## Data Availability

The datasets analysed during the current study come from the national emergency preparedness registry for COVID-19 (Beredt C19), housed at the Norwegian Institute of Public Health. The preparedness registry comprises data from a variety of central health registries, national clinical registries, and other national administrative registries. Legal restrictions prevent the researchers from sharing the dataset used in the study. However, external researchers are freely able to request access to linked data from the same registries from outside the structure of Beredt C19, as per normal procedure for conducting health research on registry data in Norway (https://www.helsedata.no). Further information on the preparedness registry, including access to data from each data source, is available at https://www.fhi.no/en/id/infectiousdiseases/coronavirus/emergency-preparedness-register-for-covid-19/ Code and model results in summary form from R is available from the author.

## Abbreviations

Beredt C19: Norwegian National Preparedness Register for COVID-19
CI: Confidence interval
DÅR: Cause of Death Register
ICU: Intensive care unit
MSIS: The Norwegian Surveillance System for Communicable Diseases
NIPaR: The Norwegian Intensive Care and Pandemic Registry
NPR: National Population Registry
SSB: Statistics Norway
SYSVAK: The Norwegian Immunisation Register
